# How schools can help to build healthy, productive lives, free of trachoma

**Published:** 2017-08-07

**Authors:** Jaouad Hammou, Gardachew Tiruneh, Abebaw Kebede

**Affiliations:** 1 National Coordinator of the Prevention of Blindness Programme, Morocco.; 2 CARE Ethiopia, WASH-Learning, Design & Measurement Advisor.; 3 CARE Ethiopia; WaTER+ Programme Coordinator.

**Children can be effective behaviour-change ambassadors and schools can act as key sites for health interventions to combat trachoma, especially when awareness forms part of the curriculum. These examples from Morocco and Ethiopia illustrate the important role that schools can play in efforts to end trachoma.**

**Figure:**
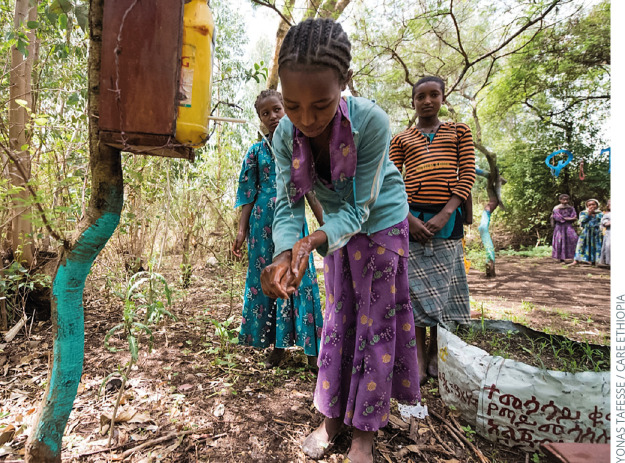
Young girls practising good hygiene in the South Gondar zone. ETHIOPIA

Children are particularly vulnerable to ocular *C. trachomatis* infection, which causes trachoma. The bacteria can spread easily to siblings and playmates via flies, towels and dirty hands. Good hygiene and facial cleanliness form part of the World Health Organization (WHO) SAFE strategy: Surgery to correct trichiasis, Antibiotics to reduce active infection, and Facial cleanliness and Environmental improvements to reduce transmission. School-based trachoma interventions provide an opportunity to raise children's awareness of the importance of hygiene. Typically, these interventions involve students learning about the causes and consequences of trachoma and the behaviours they can adopt to avoid contracting the disease. They then, in turn, disseminate these messages further amongst their families and community members.

Morocco was one of the first countries to implement the comprehensive SAFE strategy at scale. Progress was rapid, thanks in large part to strong partnerships and government buy-in at all levels, including the Ministry of Education. The effectiveness of school outreach strategies is dependent on the proportion of school-aged children who attend school. When the national programme to eliminate trachoma was rolled out in the late 1990s, Morocco benefited from school attendance rates exceeding 99%, which made schools excellent sites for tackling trachoma. At first, they were used for administering antibiotic treatments to treat infection, and later schools provided a platform for disseminating eye health messages, with lessons on trachoma becoming part of the national curriculum and students acting as behaviour-change ambassadors.

**Further information**

To find out more about trachoma and schools, read *Trachoma prevention through school health curriculum development* available in the ICTC resource library and online at: **www.trachomacoalition.org/resources/guide-trachoma-prevention-through-school-health-curriculum-development**

In Ethiopia, a country which is making huge progress in trachoma elimination but still carries the heaviest burden of disease, partners are harnessing the potential of schools using similar approaches to those used in Morocco. Due to investment in water and sanitation and increasing collaboration between the trachoma community and WASH sectors, the majority of endemic districts in Ethiopia have plans to deliver the full SAFE strategy. These plans include reaching children and parents with targeted messages through schools. In the mid-term review of the programme in South Gondar, signs of significant improvement in face washing practices are emerging. As of December 2016, 86% of pupils were washing their faces more than once a day compared with 13% in May 2015. 77% were using soap for face washing compared with 26% over the same period. These increases in face washing practices in schools are significant as hygiene behaviours are notoriously difficult to change, particularly in areas of water scarcity.

Key to the success in South Gondar was the development of school hygiene and sanitation clubs. These clubs appoint student ambassadors to promote hand/face washing, environmental sanitation and the proper use of latrines among their peers. In addition, a 25 litre can of water fitted with a tap in a ‘school hygiene corner’ ensures students are able to wash their faces when needed. Health extension workers are also using schools to raise awareness of good hygiene and sanitation practices within the school and the wider community through displays illustrating model health practices.

The elimination of trachoma both depends upon and supports progress in education. When integrated into the school curriculum, more children accessing education increases the chance that they will be exposed to eye health messages about trachoma prevention. At the same time, improvement in child health increases the likelihood that children will access education.

These examples from Morocco and Ethiopia illustrate the important role that schools can play in efforts to end trachoma and save future generations from contracting this potentially debilitating disease, thereby improving their chances of leading healthy, productive lives.

